# Determinants of antenatal care attendance among women residing in highly disadvantaged communities in northern Jordan: a cross-sectional study

**DOI:** 10.1186/s12978-018-0542-3

**Published:** 2018-06-07

**Authors:** Heba H. Hijazi, Mohammad S. Alyahya, Amer M. Sindiani, Rola S. Saqan, Abdulhakeem M. Okour

**Affiliations:** 1 Department of Health Management and Policy, Faculty of Medicine, Jordan University of Science and Technology, P.O. Box 3030, Irbid, 22110 Jordan; 2 Department of Obstetrics and Gynecology, Faculty of Medicine, Jordan University of Science and Technology, P.O. Box: 3030, Irbid, 22110 Jordan; 3 Department of Pediatrics and Neonatology, Faculty of Medicine, Jordan University of Science and Technology, P.O. Box: 3030, Irbid, 22110 Jordan; 4 Department of Public Health and Community Medicine, Faculty of Medicine, Jordan University of Science and Technology, P.O. Box: 3030, Irbid, 22110 Jordan

**Keywords:** Utilization, Antenatal care, Disadvantaged communities, Poverty, Experience of care, Quality, Jordan

## Abstract

**Background:**

One of the major reproductive health challenges among disadvantaged populations is to provide pregnant women with the necessary antenatal care (ANC). In this study, we suggest applying an integrated conceptual framework aimed at ascertaining the extent to which attendance at ANC clinics may be attributed to individual determinants or to the quality of the care received.

**Methods:**

Using a cross-sectional design, data were collected from a sample of 831 women residing in nine sub-districts in three northern governorates of Jordan and designated according to national categorization as persistent poverty pockets. All of the sampled women were recruited from public maternal and child health centers and interviewed using a structured pre-tested survey. This tool covered certain predictors, ranging from the user’s attributes, including predisposing, enabling, and need factors, to the essential components of the experience of care. These components assessed the quality of ANC in terms of five elements: woman–provider relations, technical management, information exchange, continuity of care, and appropriate constellation of services. Adequate ANC content was assessed in relation to the frequency of antenatal visits and the time of each visit.

**Results:**

The results of multivariate logistic regression analyses show that the use of ANC facilities is affected by various factors related to the quality of service delivery. These include receiving information and education on ANC during clinic visits (OR = 9.1; 95% CI = 4.9–16.9), providing pregnant women with opportunities for dialogue and health talks (OR = 7.2; 95% CI = 4.1–12.8), having scheduled follow-up appointments (OR = 6.5; 95% CI = 3.5–12.0), and offering dignified and respectful care (OR = 5.7; 95% CI = 2.5–13.1). At the individual level, our findings have identified a woman’s education level (OR = 1.2; 95% CI = 1.1–1.3), desire for the pregnancy (OR = 1.7; 95% CI = 1.1–2.7), and living in a district served by an ANC clinic (OR = 4.3; 95% CI = 2.3–8.1) as determinants affecting ANC utilization.

**Conclusion:**

Taking women’s experiences of ANC as a key metric for reporting the quality of the care is more likely to lead to increased utilization of ANC services by women in highly disadvantaged communities. Our findings suggest that the degree to which women feel that they are respected, informed, and engaged in their care has potential favorable implications for ANC.

## Plain English summary

Millions of women in developing countries are more likely to experience life-threatening and pregnancy-related complications because of a lack of access to adequate and good-quality antenatal care (ANC). Indicators of adequate care, as recommended by the World Health Organization (WHO), include providing pregnant women with four antenatal visits, of which the initial contact should be scheduled during the first trimester of pregnancy. In Jordan, remarkable progress has been made to expand the coverage of ANC; however, disparities and inequity in access to quality ANC services among different socioeconomic groups are still evident and subject to debate. Thus, our study aims to explore barriers that prevent women who live in low-income settings from utilizing antenatal services adequately.

This study collected quantitative data from women aged 15–49 years residing in highly disadvantaged sub-districts in three northern governorates of Jordan. Data were gathered by means of a face-to-face interview using a health facility-based survey.

Among the 831 enrolled women, 36.6% had inadequate attendance at ANC services. Our findings indicate that utilization of ANC is embedded in the context of the healthcare delivery system, in which women’s access to the necessary health education and engagement in one-to-one consultations were found to be significant predictors. Treating women with respect and dignity and providing them with scheduled follow-up appointments were also associated with ANC attendance.

As a result, to achieve significant changes in the delivery of ANC, it is extremely important for health authorities to pay more attention to the woman–provider interaction in increasing utilization of ANC and enhancing women’s experience of pregnancy.

## Background

ANC is a key strategy for reducing maternal morbidity and mortality directly by affording increased chances of the timely identification of high-risk pregnancies [1–3]. It also represents an entry point for the integrated use of skilled health personnel [4]. Empirical studies of preventive services have often found that regular monitoring of women during pregnancy is vital to reduce birth-related complications, provide supportive care, and promote safer motherhood [5, 6]. In contrast, low health service utilization throughout the prenatal period breaks the critical link in the continuum of care and contributes to poor birth outcomes [7].

In low- and middle-income countries (LMICs), ANC utilization has increased since the introduction of the 2002 WHO ANC model, known as ‘focused’ ANC (FANC) [8]. This model aims at delivering ‘reduced but goal-orientated’ clinic visits, at which essential interventions should be provided to pregnant women at specified intervals. With the FANC model, healthy women with no underlying pregnancy complications should be scheduled a minimum of four ANC visits, and more than four in the case of danger signs or pregnancy-related illnesses. For many of the essential interventions in FANC, it is crucial to initiate the care during the first trimester of pregnancy (up to 12 weeks of gestation), and schedule the second visit at 24 to 28 weeks of gestation and the third and fourth visits at 32 weeks and between 36 and 38 weeks of gestation, respectively [8, 9].

During the last decade, considerable research attention has been paid to determining the routine number of visits that are necessary to optimize the health of mothers and babies and enhance safety during pregnancy. Recently, however, a 2015 systematic review of randomized controlled trails has raised concerns that the reduced number of antenatal contacts is associated with an increased risk of perinatal mortality, particularly stillbirth [10]. Accordingly, in November 2016, the WHO began promoting a new model of ANC aimed at reducing perinatal deaths and improving women’s experience of care by recommending a minimum of eight contacts [8].

While many may argue that increasing the frequency of antenatal visits would positively influence the health of mothers and newborns [8, 10], related literature has indicated that a reduced number of visits, but with targeted interventions at each visit, proved to be equally effective as monthly ANC visits [11]. In many resource-limited settings, increasing the number of ANC visits for women with uncomplicated pregnancies to more than four has not been found to be associated with improved birth outcomes [12, 13]. Considering that the optimum number of ANC visits in low-income settings depends not only on effectiveness but also on feasibility and other barriers to ANC access and supply [7], our study placed particular emphasis on investigating the delivery of ‘reduced and goal-orientated’ ANC.

In Jordan, only a few studies have examined factors that predict the utilization of ANC services. Importantly, previous research has generally focused on studying the association between the use of ANC facilities with a range of individual characteristics, such as a woman’s age, occupation, parity, and level of education [14–17]. However, this may be imprecise and offers little insight into the context in which utilization occurs. Other attributes, including the accessibility, availability, and quality of ANC services, are also significant factors to consider. Hence, the main objectives of our study are to explore barriers that prevent women who live in highly disadvantaged communities from utilizing ANC services adequately, and to ascertain the extent to which their attendance may be attributed to the quality of the ANC service delivery or to the individual determinants of these women. Addressing such barriers is considered a matter of equality, social justice, and ethics, requiring policymakers to think differently about the poor and those at risk of social exclusion.

### Jordan overview

The current national coverage rate for ANC is high in Jordan; almost all women (99%) receive ANC from medically trained personnel (doctors, nurses, or midwives) at least once during pregnancy [18]. While it is obvious that Jordan is on track to achieving its goal of expanding coverage of ANC services, most efforts to enhance the use of these services have focused on quantifiable issues, such as increasing the number of prenatal visits. Data on ANC are obtained mostly from household surveys, in which women who have had a live birth during the last five years preceding the survey are asked whether and from whom they received care. However, no information on the quality of ANC services is provided by the national surveys. Further, no specific data on ANC utilization are available for those who live in highly disadvantaged communities (i.e., poverty pockets).

To facilitate the geographic targeting of the poor, Jordan has developed a system for mapping poverty, using the Small Area Estimation Method to identify ‘poverty pockets’ across the country [19]. A poverty pocket represents a district or sub-district, which may be a village, a desert oasis, or a large, low-income locality within a metropolitan city or in the suburbs, where more than 25% of the population is below the poverty line [19]. Based on the Household Expenditure and Income Surveys (HEIS) data, most recently updated in 2010/2011, the absolute poverty line amounted to 814 Jordanian Dinars (JDs) annually or 67.8 JD per capita per month [19]. This measure continues to be used as the principal measure of poverty in Jordan, and the majority of the poor are still clustered around this line [19].

During the last decade, Jordan has made strenuous efforts in fighting poverty, within its limited resources and in the context of the global political and economic upheavals. However, figures released by the Ministry of Planning and International Cooperation (MoPIC), with the technical support of the United Nations Development Program (UNDP), show an increase in poverty rates from 13.3% in 2008 to 14.4% in 2010 [20]. The number of poverty pockets also increased from 22 pockets in 2006 to 32 and 36 poverty pockets in 2008, 2010, respectively [20].

Another worrisome finding reported by the United Nations Children’s Fund (UNICEF) in 2016 is that a notable variation in the source of services across all social classes is apparent in Jordan. Women from lower wealth quintiles represent a larger share of women who access ANC services and deliver their babies at public facilities [21]. According to the National Strategy for the Health Sector in Jordan for the years 2015–2019, there are significant disparities with respect to the quality of health services between the health sector institutions and between different geographic regions [22]. This raises concerns about equitable access to quality ANC services among different socioeconomic groups within the country.

### Conceptual framework

A woman’s decision to seek ANC is not simply a matter of personal preference; the accessibility of services that either facilitate or impede utilization also has an important influence [23]. As outlined by the WHO, access to ANC services consists of several elements, including distance and/or time to a facility, the physical availability of services, cultural and social factors that may impede access, economic and other costs associated with use of services, and the quality of the services offered [24].

In a healthcare context, the use of services is a complex human behavioral phenomenon [25]. It reflects a point at which patients’ needs meet the professional system [26]. During the last few decades, a number of models have been developed to identify factors affecting the utilization of healthcare services, the most widely used being the 1995 version of the Andersen’s behavioral model [26]. According to this model, an individual’s use of a service is considered to be a function of three components. These include predisposing factors, which represent the sociocultural characteristics of individuals that exist prior to their illness or condition; enabling factors in terms of the logistical aspects of obtaining care; and need factors that generate the necessity for healthcare services, such as the presence of diagnosed medical conditions and self-perception of health status.

Despite the importance of individual determinants affecting the utilization of care, it is well known that service attendance is strongly dependent on the quality of care experienced at healthcare facilities [26]. The philosophy behind providing pregnant women with FANC is to ensure access to goal-oriented and high-quality care for all women, not only those at risk, with targeted interventions at each visit [11]. Achieving improvements in the quality of ANC is multidimensional and requires increased attention to the process of care delivery, in which the experience of care should be evaluated based on globally determined and locally feasible criteria. In 2016, the WHO released a global framework for improving the quality of care throughout pregnancy, childbirth, and the postnatal period, quality being understood as the provision and experience of care from a health systems perspective [27].

Recent evidence has acknowledged that, if the quality of ANC is poor and women’s experience is negative, women will not attend ANC services, regardless of the recommended number of contacts in the model [8, 28]. Accordingly, our study provides an additional consideration for identifying gaps in the experience of care that may lead to poor delivery of adequate care. Within the WHO framework, the experience of care consists of effective communication between healthcare providers with pregnant women; care that involves respect and the preservation of dignity; and access to social and emotional support that is sensitive to women’s needs [27].

**Fig. 1:**
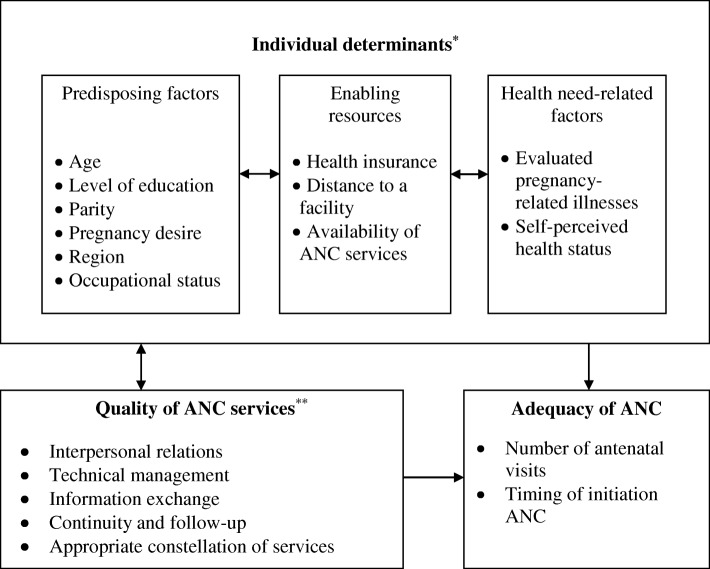
Conceptual framework for analyzing determinants of ANC utilization among women residing in highly disadvantaged communities in northern Jordan *Adapted from Andersen’s behavioral model for health service use (Andersen, 1995) **Adapted from Bruce's framework for assessing the quality of care (Bruce, 1990)

Inspired by the 2016 WHO framework and taking into account the study’s main objectives, we suggest applying an integrated conceptual framework that incorporates both individual determinants for seeking care and the contextual characteristics of ANC service delivery. Specifically, our study aims to explore the effects of quality of care on the utilization of ANC and how the experience of care can lead to different access outcomes for women from lower-income backgrounds. As per Fig. 1, individual determinants are categorized as predisposing, enabling, and need factors; while variables related to the quality of ANC capture the essential components of the experience of care.

To assess the quality of ANC experienced, our study is directed by Bruce’s framework for conceptualizing the quality of care in the provision of reproductive health services [29]. Within this framework, evaluating the quality of ANC requires the examination of five main elements, namely interpersonal relations between healthcare providers and clients; technical management; information exchange; continuity and follow-up; and an appropriate constellation of services [29].

## Methods

### Study design and sample

This study collected quantitative data from 14 sub-districts, identified by the MoPIC as poverty pockets, in northern Jordan. These sub-districts are spread over three northern governorates (the administrative division in the country), Irbid, Mafraq, and Jerash, and classified as rural and desert towns/villages. The modeling of the 14 sub-districts is compatible with the sampling frame of the HEIS 2011 and the social data pilot module survey launched by MoPIC and UNDP to measure vulnerability risk and social exclusion in the country. Over the last decade, nine of these sub-districts are classified as persistent poverty pockets, and five are classified as fluctuating ones [20]. To ensure that all poverty pockets shared the same characteristics, only persistent ones were included in our study. Furthermore, all of the sampled sub-districts are broadly similar in their level of socioeconomic wealth (i.e. sanitation, electricity, and water service) and age/gender structure.

Out of the nine selected sub-districts, five were served by public Maternal and Child Health (MCH) centers; however, the remaining four had no such service. Given that the target population of this study was users of reproductive health services at public MCH centers, and that these centers provide services to well-defined geographic areas, the nearest center in each of the underserved sub-districts was targeted. Before starting the fieldwork, the names of MCH centers were obtained using a list provided by the Jordanian Ministry of Health (MoH). During this phase, new maps of all sub-districts were prepared, and the numbers of MCH centers were updated, listed, and documented.

The field work stage of data collection started in June 2015 and ended in July 2016. Using purposive sampling, efforts were made to ensure that all participating women met a number of inclusion criteria. In particular, a woman was eligible to participate only if she was a currently married woman, aged 15–49 years, had been living in one of the chosen highly disadvantaged sub-districts for at least the preceding four years, and had a household monthly income of 68 JDs or less per person and was receiving no other type of assistance. The primary users of services provided at the public MCH centers are married women of childbearing age from the middle and low socioeconomic classes, and thus most of the facilities’ attendees were eligible for participation. Since the main focus of this study was to assess the quality of care as perceived by the women receiving the care, a number of exclusion criteria were applied. These included any currently married woman within the specified age group who had not had a live birth in the last two years preceding the survey (*n* = 136), was pregnant at the time of the survey (*n* = 118), did not have at least one ANC visit during the last pregnancy (*n* = 21), initiated ANC visits after the first trimester (*n* = 190), and obtained ANC from multiple/other facilities, or was a new registry (*n* = 215). Of the total 1511 women interviewed in the survey, with a 96% response rate, data from 831 participants were eligible for analysis.

### The study instrument and variables

#### Data collection instrument

This study is based on original cross-section data collected by means of a face-to-face interview using a structured, pre-tested survey. The survey was adopted after reviewing the literature in the relevant research area. Prior to data collection, several steps were taken to assess the validity and reliability of the instrument. To examine content validity, a panel of eight experienced perinatal personnel was asked to review and refine each item in the instrument. This panel was composed of three obstetricians, three senior community midwives, and two researchers in the field of reproductive health. Further, the Pearson correlation coefficient was calculated to examine the test–retest reliability of the survey for a sub-sample of participants (*n* = 28). The questionnaire was administered again to the same sample three weeks later. The test–retest correlation coefficient for the instrument was 0.78 (*p* < 0.001), which is indicative of acceptable stability over time.

#### Outcome variable

In our analysis, the utilization of ANC services is classified into adequate versus inadequate as the outcome variable. To assess the overall adequacy of ANC, reviews of the medical records of respondents were conducted to obtain more accurate information about the basic ANC components received (i.e., frequency of antenatal visits and timing of each visit). Keeping in mind that this study was limited to the sample of women who initiated antenatal visits during the first trimester, ANC was defined as adequate if the woman with a normal pregnancy had at least four antenatal visits and more than four in case of complications; otherwise, it was defined as inadequate.

#### Explanatory variables

The study’s explanatory variables consist of a number of covariates and predictors. Based on the previous conceptual framework and a review of the literature, several covariates were taken into consideration. These covariates embodied the background determinants of respondents: predisposing characteristics (i.e., age, education, parity, occupation, desire for the pregnancy, and region); enabling resources (i.e., health insurance, living in an area served by a MCH center, and the time taken to get to the nearest center); and need factors (i.e., evaluated pregnancy-related illnesses and self-perceived health status). Using a list of pregnancy-related complications, the participating women were asked to self-report the main illnesses that they had had during their last pregnancy. This list was identified by obstetricians and involved a number of medical conditions, including hypertension, diabetes, anemia, respiratory distress, abnormal vaginal discharge, pre-eclampsia, joint problems, and urinary tract infection.

Factors related to the perceived quality of ANC were included in the analysis as the main predictors of interest. These involved interpersonal relations (i.e., communication, understanding, and the presence of privacy); technical management (physical examination, availability of vaccines/supplements, and clinical tests); information exchange (clarity of providers’ explanations, consultation time, and obtaining information and counseling on ANC); follow-up and continuity (i.e., scheduling of appointments and the sincerity of the provider); and an appropriate constellation of services (i.e., waiting time and opening hours).

## Statistical analyses

Summary statistics were carried out to describe the study participants according to different characteristics. Differences in ANC attendance (i.e., adequate vs. inadequate use) were reported using chi-squared tests and two sample t-tests for categorical and continuous variables, respectively. Standardized residuals were examined for categorical variables to determine which cell contributed to the statistically significant difference between the observed frequency and the expected one. Binary logistic regression models were performed to obtain adjusted odds ratios (ORs) for the outcome variable, using the SPSS 20.0 statistical package. Statistical significance was set at *p* < 0.05.

## Results

### Descriptive analysis

Analyses for characteristics of mothers using ANC services are presented in Table 1. The ages of the women at the birth of their last child ranged from 16 to 45 years, with a median age of 31 years. Forty-eight percent had received 10 or fewer years of schooling. The average total number of living children was 3.6, giving a range between 1 and 12 children. The majority of the respondents was housewives (85.4%) and had public sector insurance coverage (69.8%). The health status of 48.5% of participants was average during the last pregnancy, and 33.6% of the sample had had a high-risk pregnancy. Urinary tract infection, joint problems, and anemia were the three most common pregnancy-related illnesses among respondents, respectively. Concerning ANC attendance, 63.4% of women received the recommended care components, while 36.6% received inadequate ANC.

**Table 1:** Baseline characteristics of the sample population (*N* = 831)

Variable	n (%)	Mean; SD
Age (years)		30.99; 7.47
≤ 23	185 (22.3)	
24–31	293 (35.3)	
32–39	229 (27.6)	
≥ 40	124 (14.9)	
Education (years)		10.41; 2.71
≤ 6	69 (8.3)	
7–9	240 (28.9)	
10–12	430 (51.7)	
> 12	92 (11.1)	
Parity		3.56; 2.06
1	154 (18.5)	
2–3	284 (34.2)	
4–5	254 (30.6)	
≥ 6	139 (16.7)	
Region		–
Irbid	213 (25.6)	
Jerash	170 (20.5)	
Mafraq	448 (53.9)	
Employment status		–
Unemployed	710 (85.4)	
Employed	121 (14.6)	
Health insurance		–
Uninsured	251 (30.2)	
Insured	580 (69.8)	
Total number of pregnancy-related illnesses		–
No health problems	380 (45.7)	
1–2 health problems	172 (20.7)	
≥ 3 health problems	279 (33.6)	
Self-perceived health status		–
Poor	208 (25.0)	
Average	403 (48.5)	
Excellent	220 (26.5)	

### Differences in the usage of antenatal care services

Table 2 presents a comparative analysis of the differences between women who received the recommended basic ANC service components and those who did not, by individual determinants. Overall, there were statistically significant associations between all of the individual determinants and ANC attendance, except for employment and health status variables (*p* = 0.644 and *p* = 0.837, respectively). On bivariate analysis, the means of age and the number of living children variables were significantly lower among women who attended ANC adequately. Being more educated was also found to be positively associated with receiving adequate ANC services. Likewise, the proportions of women who had adequate use of ANC services were significantly higher among those who had wanted the pregnancy (67.6%), resided in Irbid governorate (72.8%), were insured (68.1%), lived in a neighborhood served by MCH services (71%), required less than 15 min to get to the nearest center (68.9%), and had a high-risk pregnancy (70.3%).

**Table 2:** Comparison between adequate and inadequate utilization of ANC by individual determinants

Variables	Utilization of ANC		t-test/chi2	*p*-value
	Inadequate *n* = 304 n (%)	Adequate *n* = 527 n (%)		
Predisposing Characteristics				
Age in years^a^ (Mean; SD)	(32.49; 7.04)	(30.12; 7.58)	4.45	<0.001
Years of education^a^ (Mean; SD)	(9.69; 2.51)	(10.82; 2.73)	-5.91	<0.001
Parity^a^ (Mean; SD)	(3.97;2.05)	(3.33; 2.04)	4.33	<0.001
Desire for the pregnancy				
Unwanted	128 (44.4)	160 (55.6)	11.74	0.001
**Wanted**	176 (32.4)	367 (67.6)		
Region				
**Irbid**	58 (27.2)	155 (72.8)	17.33	<0.001
Jerash	54 (31.8)	116 (68.2)		
Mafraq	192 (42.9)	256 (57.1)		
Employment status				
Unemployed	262 (36.9)	448 (63.1)	0.21	0.644
Employed	42 (34.7)	79 (65.3)		
Enabling Resources				
Health insurance				
Uninsured	119 (47.4)	132 (52.6)	18.17	<0.001
**Insured**	185 (31.9)	395 (68.1)		
Time taken to get to the nearest MCH center in minutes				
**< 15 min**	122 (31.1)	270 (68.9)	24.06	<0.001
15 -30	123 (36.6)	213 (63.4)		
>30	59 (57.3)	44 (42.7)		
Availability of ANC services				
Underserved	155 (48.7)	163 (51.3)	32.83	<0.001
**Served**	149 (29.0)	364 (71.0)		
Health Needs				
Total number of pregnancy-related illnesses				
No health problems	153 (40.3)	227 (59.7)	8.48	0.014
1-2 health problems	68 (39.5)	104 (60.5)		
**≥ 3 health problems**	83 (29.7)	196 (70.3)		
Self-perceived health status				
Poor	74 (35.6)	134 (64.4)	0.36	0.837
Average	146 (36.2)	257 (63.8)		
Excellent	84 (38.2)	136 (61.8)		

^a^t-test was run on the following variables: age, education, and parity

Bold text represents categories that are over-represented, indicating that they were more likely to utilize ANC services adequately

Differences in the overall ANC service adequacy were also observed by a number of factors related to the quality of care. As per Table 3, the highest percentages of adequate ANC attendance were among women who were often treated in a humane, respectful, supportive environment (82.7%) and those who were able to discuss their health problems/concerns with healthcare providers (81.0%). However, maintaining privacy during consultations was not significantly associated with the outcome variable (*p* = 0.880).

**Table 3:** Comparison between adequate and inadequate utilization of ANC by factors related to the quality of service delivery

Variables	Utilization of ANC		chi2	*p*-value
	Inadequate *n* = 304 n (%)	Adequate *n* = 527 n (%)		
Interpersonal relations				
Dignified and respectful care was offered by healthcare providers				
Rarely	157 (62.5)	94 (37.5)	141.42	<0.001
Sometimes	77 (43.8)	99 (56.3)		
**Often**	70 (17.3)	334 (82.7)		
Providers were ready to discuss health problems/concerns with women				
Rarely	171 (58.6)	121 (41.4)	105.85	<0.001
Sometimes	70 (33.8)	137 (66.2)		
**Often**	63 (19.0)	269 (81.0)		
Privacy was maintained during consultations				
Rarely	94 (37.5)	157 (62.5)	0.26	0.880
Sometimes	36 (34.6)	68 (65.4)		
Often	174 (36.6)	302 (63.4)		
Technical management				
Comprehensive physical exams were performed				
Rarely	117 (50.4)	115 (49.6)	63.68	<0.001
Sometimes	105 (46.7)	120 (53.3)		
**Often**	82 (21.9)	292 (78.1)		
Vaccines/supplements were available at the facility				
Rarely	108 (39.6)	165 (60.4)	1.81	0.404
Sometimes	36 (33.0)	73 (67.0)		
Often	160 (35.6)	289 (64.4)		
Clinical tests were routinely conducted				
No	196 (36.8)	337 (63.2)	0.02	0.879
Yes	108 (36.2)	190 (63.8)		
Information exchange				
Information received on ANC was				
Little	175 (61.2)	111 (38.8)	171.19	<0.001
Fair	80 (46.8)	91(53.2)		
**Enough**	49 (13.1)	325 (86.9)		
Provider explanations were easily to understand				
Rarely	86 (65.6)	45 (34.4)	78.85	<0.001
Sometimes	68 (48.2)	73 (51.8)		
**Often**	150 (26.8)	409 (73.2)		
Consultation time was				
Short	136 (60.7)	88 (39.3)	101.62	<0.001
Average	78 (42.4)	106 (57.6)		
**Enough**	90 (21.3)	333 (78.7)		
Continuity and follow-up				
Follow-up appointments were regularly scheduled by providers				
Rarely	180 (61.2)	114 (38.8)	157.43	<0.001
Sometimes	78 (40.2)	116 (59.8)		
**Often**	46 (13.4)	297 (86.6)		
A return visit was encouraged by healthcare providers				
Rarely	123 (55.4)	99 (44.6)	46.95	<0.001
Sometimes	23 (34.3)	44 (65.7)		
**Often**	158 (29.2)	384 (70.8)		
Appropriate constellation of services				
Time had to wait was				
Short	21 (33.9)	41 (66.1)	0.22	0.894
Average	124 (37.0)	211 (63.0)		
Long	159 (36.6)	275 (63.4)		
Hours of service were				
Inconvenient	76 (38.0)	124 (62.0)	0.23	0.633
Convenient	228 (36.1)	403 (63.9)		

Bold text represents categories that are over-represented, indicating that they were more likely to utilize ANC services adequately

In relation to the technical management features, no significant differences were noticed between the two groups of interest, with the exception of the physical examination variable. Women who reported that a comprehensive physical exam was often performed at the center represented the highest rate of adequate ANC attendees (78.1%). Availability of vaccines/supplements at the facility and conducting clinical tests during the antenatal period were not found to be significantly associated with the utilization of ANC (*p* = 0.404 and *p* = 0.879, respectively).

It is also interesting to note that there were statistically significant differences in the adequacy of ANC in terms of information exchange. In particular, the proportion of women who utilized ANC adequately was significantly higher among those who reported that the procedures and the diagnosis were often explained clearly by providers (73.2%), received enough information about ANC (86.9%), and had enough consultation time (78.7%).

By continuity and follow-up variables, the highest percentages of adequate ANC users were among women who often had follow-up appointments scheduled by health workers (86.6%), and those who reported that they were encouraged by providers to make a return visit (70.8%). In contrast, no significant differences in the utilization of care were observed according to the appropriateness of the constellation of services, such as waiting time and service hours (*p* = 0.894 and *p* = 0.633, respectively).

### Predictors of antenatal care attendance

Following the study’s objectives, three models were employed to estimate the association between the explanatory variables (i.e., covariates and predictors) and the response variable (i.e., adequacy of ANC attendance). Table 4 summarizes the logistic regression estimation results for the three models. Each factor showing a statistically significant association with the response variable was interpreted controlling for the effects of other explanatory variables included in the model.

**Table 4:** Multivariate regression analysis predicting the utilization of ANC among women in highly disadvantaged communities in northern

Variables	Individual determinants (Model 1)	Quality of service delivery (Model 2)	Both (Model 3)
	Adjusted OR (95% CI)	Adjusted OR (95% CI)	Adjusted OR (95% CI)
Predisposing factors			
Age in years	0.98 (0.95-1.01)		0.98 (0.93-1.03)
Years of education	**1.15** ^**^ **(1.09-1.23)**		**1.16** ^*^ **(1.06-1.27)**
Parity	0.94 (0.84-1.05)		0.92 (0.77-1.09)
Desire for the pregnancy: (unwanted)			
Wanted	**1.82** ^**^ **(1.31-2.52)**		**1.70** ^*^ **(1.06-2.72)**
Region: (Irbid)			
Jerash	0.92 (0.57-1.51)		1.73 (0.86-3.51)
Mafraq	**0.48** ^*^ **(0.29-0.77)**		0.61 (0.30-1.23)
Employment status: (unemployed)			
Employed	0.80 (0.51-1.26)		1.45 (0.75-2.81)
Enabling recourses			
Health insurance: (uninsured)			
Insured	0.81(0.50-1.32)		0.72 (0.35-1.48)
Time taken to get to the nearest MCH center in minutes: (< 15 min)			
15-30	0.92 (0.66-1.29)		0.97 (0.60-1.56)
≥30	**0.42** ^*^ **(0.26-0.69)**		**0.35** ^*^ **(0.17-0.70)**
Availability of ANC services: (underserved)			
Served	**2.73** ^**^ **(1.85-4.03)**		**4.34** ^**^ **(2.34-8.07)**
Health needs			
Total number of pregnancy-related illnesses: (no health problems)			
1-2 health problems	1.01 (0.67-1.50)		0.80 (0.45-1.44)
≥ 3 health problems	**2.14** ^**^ **(1.47-3.13)**		1.66 (0.97-2.84)
Self-perceived health status: (poor)			
Average	1.03(0.70-1.51)		1.48 (0.85-2.59)
Excellent	0.94(0.61-1.44)		1.41 (0.76-2.60)
Patient-provider relations			
Dignified and respectful care was offered by healthcare providers: (rarely)			
Sometimes		2.09 (0.61-7.08)	1.80 (0.43-7.52)
Often		**5.54** ^**^ **(2.60-11.81)**	**5.68** ^**^ **(2.45-13.13)**
Providers were ready to discuss health problems/concerns with women: (rarely)			
Sometimes		**2.37** ^*^ **(1.41-3.97)**	**2.28** ^*^ **(1.27-4.07)**
Often		**5.38** ^**^ **(3.28-8.82)**	**7.22** ^**^ **(4.06-12.82)**
Privacy was maintained during consultation: (rarely)			
Sometimes		0.56 (0.12-2.63)	0.46 (0.09-2.52)
Often		0.70 (0.21-2.25)	0.38 (0.09-1.58)
Technical management			
Comprehensive physical exams were performed: (rarely)			
Sometimes		1.08 (0.61-1.93)	1.19 (0.61-2.33)
Often		1.45 (0.82-2.54)	1.58 (0.84-2.97)
Vaccines/supplements were available at the facility: (rarely)			
Sometimes		1.86 (0.41-8.43)	2.24 (0.43-11.82)
Often		1.87 (0.58-5.99)	3.63 (0.89-14.83)
Clinical tests were regularly conducted: (no)			
Yes		1.29 (0.84-1.97)	1.27 (0.77-2.08)
Information exchange			
Information received on ANC was: (little)			
Fair		1.11 (0.56-2.22)	1.16 (0.54-2.51)
Enough		**7.34** ^**^ **(4.26-12.66)**	**9.12** ^**^ **(4.91-16.92)**
Consultation time was:(short)			
Average		1.04 (0.29-3.69)	1.11 (0.26-4.81)
Enough		0.65 (0.29-1.46)	0.51 (0.21-1.27)
Provider explanations were easily to understand: (rarely)			
Sometimes		1.02 (0.53-1.96)	0.77 (0.37-1.62)
Often		**1.97** ^*^ **(1.12-3.46)**	**2.38** ^*^ **(1.22-4.66)**
Continuity and follow-up			
Follow-up appointments were regularly scheduled by providers: (rarely)			
Sometimes		**3.03** ^*^ **(1.46-6.29)**	**3.65** ^*^ **(1.61-8.28)**
Often		**6.05** ^**^ **(3.51-10.43)**	**6.51** ^**^ **(3.52-12.01)**
A return visit was encouraged by healthcare providers: (rarely)			
Sometimes		1.90 (0.89-4.07)	1.66 (0.72-3.83)
Often		**2.42** ^**^ **(1.53-3.83)**	**1.82** ^*^ **(1.08-3.06)**
Appropriate constellation of services			
Time had to wait was: (short)			
Average		0.86 (0.39-1.94)	0.63 (0.25-1.57)
Long		0.68 (0.31-1.50)	0.65 (0.27-1.57)
Hours of service were: (inconvenient)			
Convenient		0.94 (0.59-1.51)	0.96 (0.56-1.63)
Nagelkerke R^2^	0.205	0.574	0.660

*p < 0.05; **p < 0.001

The reference category is in parentheses

Initially, the first model was developed to investigate which of the individual’s determinants are associated with receiving sufficient contents of ANC, and therefore only covariates were included in this model. As the figures in Table 4 indicate, the years of education showed a significant association with the use of ANC (95% CI = 1.09–1.23). For a one-year increase in women’s education, the odds of utilizing ANC services increased by a factor of 1.15. Women’s desire to become pregnant was also found to have a positive association with the use of care, in which women who had a desire to get pregnant were 1.82 times more likely to attend ANC adequately compared to those who had an unwanted pregnancy (95% CI = 1.31–2.52). According to this model, mothers residing in the governorate of Mafraq were 52% less likely to have an adequate usage of ANC services than those living in Irbid (95% CI = 0.29–0.77). Our results also illustrated that, as the travel time to the nearest center increased, the odds of reporting an adequate use of ANC declined by a factor of 0.42 (95% CI = 0.26–0.69). Women who lived in a neighborhood served by MCH services were 2.73 times more likely to report adequate use of ANC (95% CI = 1.85–4.03). In respect of health needs variables, women who had experienced three or more previous pregnancy-related illnesses were 2.14 times more likely to attend ANC adequately compared to those had had a normal pregnancy (95% CI = 1.47–3.13).

To ascertain the extent to which the utilization of ANC services may be attributed to the quality of the ANC service delivery, only the predictors of interest were included in the second model. The results of this model demonstrated that women who often received dignified and respectful treatment from healthcare providers were 5.54 times more likely to utilize ANC adequately compared to those who rarely had positive communication with providers (95% CI = 2.60–11.81). Additionally, the odds of reporting an adequate use of ANC services increased by factors of 2.37 (95% CI = 1.41–3.97) and 5.38 (95% CI = 3.28–8.82) for women who were sometimes and often able to discuss their health problem/concerns with healthcare providers, respectively.

According to the second model, women who received enough information and counselling on ANC were 7.34 times more likely to attend the targeted care as recommended compared to those who obtained little information (95% CI = 4.26–12.66). The odds of reporting adequate use of ANC also increased by a factor of 1.97 for women who perceived that the provider’s explanations on pregnancy-related issues were clear compared to those who had poorer explanations (95% CI = 1.12–3.46).

Analysis of factors associated with the continuity of services revealed some significant results. The odds of reporting the adequate use of ANC were increased by factors of 3.03 (95% CI = 1.46–6.29) and 6.05 (95% CI = 3.51–10.43) for women who sometimes and often had regular follow-up appointments scheduled by healthcare providers, respectively. Women who were often encouraged to make a return visit were 2.42 times more likely to utilize ANC adequately compared to those who were rarely encouraged to come back (95% CI = 1.53–3.83). In contrast, variables related to technical management and the appropriateness of the constellation of services included in the second model did not show statistically significant associations with the outcome variable (Table 4).

All of the study’s covariates and predictors were included in the third and final model, to identify if the quality of care was associated with the adequacy of ANC attendance, controlling for the influences of individual determinants. Based on this model, certain variables related to predisposing factors and enabling resources remained significant; however, health needs no longer had statistically significant associations with the response variable. As per Table 4, for a one-year increase in the woman’s education, the odds of utilizing ANC services adequately increased by a factor of 1.16 (95% CI = 1.06–1.27). Likewise, women who had wanted the pregnancy were 1.7 times more likely to attend ANC adequately than those who had an unplanned pregnancy (95% CI = 1.06–2.72). Women who lived in a neighborhood served by MCH services were 4.34 times more likely to report adequate use of ANC (95% CI = 2.34–8.07). Our analysis also revealed that, as the travel time to the nearest center increased, the odds of reporting adequate utilization of ANC declined by a factor of 0.35 (95% CI = 0.17–0.70).

The results of the final model also illustrated that dimensions related to women–provider relations, information exchange, and follow-up and continuity were all significant predictors for the utilization of ANC services. Specifically, women who often received respectful and friendly treatment from healthcare providers were 5.68 times more likely to utilize ANC adequately compared to those who rarely had positive communication with providers (95% CI = 2.45–13.13). The odds of reporting adequate use of ANC services were also increased by factors of 2.28 (95% CI = 1.27–4.07) and 7.22 (95% CI = 4.06–12.82) for women who sometimes and often had opportunities for dialogue and health talks with staff, respectively.

Women’s exposure to enough information, education, and advice on ANC during clinic visits had a significant positive association with ANC attendance (OR = 9.12; 95% CI = 4.91–16.92). Compared to mothers who reported limited understanding of providers’ explanations on issues related to diagnosis and procedures, the odds of reporting adequate utilization of care increased by a factor of 2.38 for those who often received clear explanations (95% CI = 1.22–4.66).

Women who were often encouraged by health workers to continue antenatal visits at the same facility were 1.82 times more likely to utilize ANC adequately compared to those who were rarely encouraged to come back (95% CI = 1.08–3.06). Our analysis also indicates that the odds of reporting the adequate use of ANC were increased by factors of 3.65 (95% CI = 1.61–8.28) and 6.51 (95% CI = 3.52–12.01) for women who sometimes and often had regular follow-up appointments scheduled by healthcare providers, respectively. Similarly to the second model, technical management and the appropriateness of the constellation of services were not found to be significant predictors of ANC utilization (Table 4).

## Discussion

This paper suggests applying an integrated conceptual framework to ascertain the extent to which the attendance at ANC clinics may be attributed to individual determinants among women from disadvantaged backgrounds or to the quality of service delivery. Empirical research on healthcare utilization has shown that users’ characteristics account for an estimated 20 to 25% of the variance explained [30]. This is consistent with our findings, in which individual determinants (model 1) accounted for 21% of the systematic explained variance compared to 57.4% of the variance explained by the quality of ANC (model 2). Once the two models have been merged, the variance explained by the final model increased to 66.0%. This implies that the variations in the use of ANC facilities were mainly attributable to the quality of service as experienced by women at healthcare facilities.

One of the major concerns of current research is to explore the barriers that prevent women in highly disadvantaged communities from attending ANC adequately. According to the final model, adequacy of ANC coverage is affected by a number of health service and individual factors. In particular, our findings illustrate that the increase in the woman’s level of education is a significant motivator for increasing the likelihood of her ANC attendance. This concurs with other studies demonstrating that the low level of a woman’s education is associated with infrequent and no ANC, as well as delay in accessing medical help [1, 2, 9, 12, 31]. It makes sense that educated women are more likely to appreciate the benefits of ANC for their health and their children’s well-being and to exercise autonomy and decision-making power. More years of schooling may also promote women’s ability to approach health staff to ask questions and to discuss any possible health concerns.

The actual desire of women to become pregnant was also found to be an important factor in determining the adequacy of ANC. A study by Muhwava et al. [12] also supports this finding, revealing that wanted pregnancy was associated with increased odds of early initiation and adequate ANC attendance compared to unwanted pregnancy. Another study, done in Holeta in central Ethiopia [32], showed that women who reported an unplanned pregnancy were 67% less likely to attend ANC than those who reported they had planned the pregnancy. Researchers argue that unplanned pregnancy is highly likely to reflect a lack of access to education on family planning during antenatal visits [32, 33].

Not surprisingly, women’s decision to use ANC services is affected by having the means available to utilize these services. Research findings from resource-poor sites (i.e., Malawi and Kenya) have indicated that a large distance to reach a health facility was associated with delayed initiation of ANC use and low frequency of care attendance [9]. One explanation for such behavior is women’s desire to minimize the number of journeys to the facility and thereby their total expenditure on ANC. In the case of Jordan, in spite of policies promising free ANC, many women in disadvantaged communities tend to avoid clinic visits because they are unable to afford the associated travel costs. Considering that the actual ANC is being provided free of charge, this might explain the non-significant finding of the association between health insurance and ANC attendance in the study’s models.

In the same context, our study revealed that living in a district served by an MCH clinic was a potential contributing factor to increasing the use of ANC among poor women in northern Jordan. These findings are in agreement with previous research conducted in underserved populations, where living closer to a health facility was found to be a motivator for ANC use, as absence from work and transportation costs might be minimized [34, 35].

At the health service level, our study highlights the importance of several factors in enhancing the frequency of ANC attendance, the most prominent of which is the information and education women received on ANC during one-to-one consultations. This assertion is supported by the literature, in which the provision of maternal health information by healthcare professionals has been reported to be an effective means of encouraging the use of ANC during pregnancy [9, 31]. Research has shown that improving women’s access to the necessary health education is fundamental for increasing their knowledge of ANC benefits and helping them make informed decisions about their care [1, 36]. In a study by Asfawosen et al. [37], the mother’s knowledge about pregnancy danger signs had the potential to impact positively on ANC utilization. A study by Erlindawati et al. [38] also illustrated that pregnant women who obtained enough information about ANC services had a lower percentage of inadequate utilization compared to those who received fair but limited information. In the literature, it has been reported that pregnant women with low quality of ANC and no counseling services were at risk of pregnancy complications owing to the lack of relevant information [39, 40]. According to a study by Ejigu et al. [40], receiving incomplete information about ANC was also among the reasons for mothers’ dissatisfaction with the overall perceived quality of care offered in ANC clinics in northwest Ethiopia.

Women’s attendance at ANC facilities was also found to be related to ease of understanding the provider’s explanations on pregnancy-related issues. Researchers have asserted that the limited understanding women received during their visit to a health facility represents a ‘missed opportunity’ to inform women about the possible complications of pregnancy [41–43]. The delivery of clear advice and messages that explain how to prevent and manage problems (i.e., miscarriage, multiple births, and abnormal position of the baby) can result in better recognition of the importance of attending ANC in improving health outcomes for mothers and children.

It was not a surprise to find an association between the woman–provider relationship and the use of ANC services. This link is most apparent in studies showing that building a positive rapport with pregnant women can contribute to their achieving the recommended number of antenatal visits. In contrast, researchers have pointed out that having low-quality care because of providers’ poor communication skills (which may include an abusive attitude on the part of staff) may negatively influence ANC attendance [32, 40, 44]. In the study by Birmeta et al. [32], one of the participating women stated that the reason for underutilization of ANC was that, “If you go to a health institution you will find a low quality of services and a lack of respect and mistreatment from some of the healthcare providers”. Obviously, the lack of women’s desire to return to the clinic could be largely based on their expectations about the care provided, which may depend on previous experience.

Similarly, this study demonstrated that providing pregnant women with opportunities for dialogue and health talks during visits had a central role in increasing their motivation to attend ANC adequately. Many have argued that allowing women to communicate their concerns to health staff may encourage them to start ANC earlier and to have at least four ANC visits [9]. It is also important to mention that women who have good relations with health staff may feel more supported to ask questions and talk about their reproductive concerns and problems [45].

Interestingly, our study illustrated that scheduling follow-up appointments is likely to have an impact on women’s decision about future visits. It is widely known that healthcare providers exercise significant authority and their advice is generally trusted by women. In view of that, instructions about when to attend ANC communicated by health staff apparently influence ANC utilization. Similarly, a lack of encouragement to attend upcoming appointments can result in delays to accessing ANC [9]. In Jordan, a study conducted by Al-Qutob et al. [35] found that almost 80% of the women were satisfied with ANC when the return visit was scheduled by the midwife; moreover, their satisfaction level had improved when providers explained to them the advantages of the timed follow-up visit.

### Policy considerations

#### Health education and information exchange

Our study provides additional evidence that it is critical for health authorities to put considerable efforts into enhancing the quality of ANC by providing pregnant women with proper counseling that includes supportive listening, advice giving, and relevant information. Engaging mothers as active participants in consultations would also help make changes in healthcare delivery from one based on provider-dominated dialogues to one that involves users in the decision-making process. This requires a transition in the role of health workers from one characterized by authority to one that depends on collaboration and partnership between patients and providers.

Antenatal information should also be given in a form that is easy to understand and accessible to the users of ANC services. Medical providers’ explanations of reproductive health matters should be tailored to the different social contexts, including those with low levels of education and income. Healthcare workers at MCH centers need to ascertain that the pregnant women understand the information received during consultation well enough to make an informed decision.

Since the desire to have a pregnancy was found to be a significant factor in determining adequacy of ANC attendance, it may therefore be useful to focus on informing women about effective contraceptive methods that help prevent unintended pregnancy in these populations. Promoting family planning education and more widely disseminating knowledge on contraception need to be an integrated component of ANC coverage. This research also proposes that the MoH appoint a person in each of the MCH centers to work as a reproductive health counselor. This person would be able to present clear information about birth preparedness, complications readiness, danger signs, nutrition, breastfeeding, and maternal stress.

Our study suggests using radio reports and television spots as a means of raising public awareness regarding the benefits of antenatal visits for improving babies’ and mothers’ health. Such information could provide just enough knowledge to initiate action before the women’s exposure to health education at clinics. Displaying educational posters in a prominent place at MCH centers is highly recommended.

#### Follow-up appointments and continuity of care

Given that women generally place trust in health staff’s recommendations, repeated, structured follow-up appointments and encouragement for return visits are suggested as an effective approach to improve the continuation of ANC attendance. Issuing appointment cards showing the times of upcoming ANC visits would be one possible way to remind women about the date of attendance. The relevance of this schedule also needs to be explained by health workers.

#### Women’s interpersonal relations with health staff

Recognition must be also given to the impact on women’s willingness to attend ANC of respectful and friendly treatment offered by providers. This would call for special training courses targeted at improving the communication skills of health staff on how to deal with the centers’ referents and how be more mindful, informative, and empathic. Treating women kindly and having sound interpersonal relations with them would be a key means of increasing the numbers of those turning to antenatal clinics to receive care.

It is also hugely important for healthcare providers to ensure an effective approach by giving women a chance to be heard and to encourage free discussion on their physical and psychological well-being. This study highly recommends health workers to spend more time with clients to obtain their medical histories, and to ask if they have previously experienced pregnancy-related complications. For this purpose, it is essential to increase the number of health staff working at ANC clinics.

#### Accessibility to antenatal care facilities

To achieve significant changes at the health service level, this research strongly suggests that the public sector develop health policies aimed at expanding the availability of MCH centers to cover underserved areas. Increasing the number of ANC clinics that are physically close to those who live in disadvantaged conditions would help enhance their use. Likewise, the MoH can introduce mobile ANC in order to increase utilization of perinatal services and eliminate associated travel costs.

## Conclusion

Whereas some researchers may consider the utilization of ANC services as a reflection of individual determinants, our study provides proof that this behavior is also embedded in the context of the healthcare delivery system. Specifically, our research has identified that attendance at ANC services was influenced by a number of factors, the most prominent of which are information exchange, follow-up and continuity of care, interpersonal relations with healthcare providers, and the availability of health facilities.

The results of this study have clarified that the interaction between women and providers is a key metric for the reported quality of ANC. The degree to which women feel that they are respected, informed, and engaged is considered an essential component of a successful strategy for improving mothers’ experience with ANC and a way to motivate them to attend MCH clinics. In conclusion, our study reveals the strong association between how women perceive quality of care and their decision to attend reproductive health services. Strengthening the role of healthcare providers in counseling and health education would result in greater acceptance and sustained use of ANC.

### Strengths and limitations

The potential limitations of this research were that the study may have been subject to recall bias, limited to women who were visiting the participating MCH centers at the time of the survey, and the tool did not include variables related to women’s psychosocial and cultural factors. Future studies may also try to contact women who are not attending ANC at all and those who initiate ANC late (i.e., in the second or third trimester). Considering the importance of adaptation and implementation of the new ANC model within different health systems, clinical research investigating the contribution of the updated model to increasing the likelihood of positive pregnancy outcomes should be given priority. A need for research addressing the acceptability, feasibility, and cost-effectiveness of implementing the eight-contact model in low-resource settings is also obvious.

The strengths of this study include the use of a conceptual framework that takes contextual characteristics of ANC service delivery into account, the potential impact of such factors having been little explored in literature to date. Importantly, targeting contextual factors represents an opportunity to find areas that can be improved by policy formulation and implementation, unlike individual determinants, which are difficult to change. Analyzing women’s perceptions of the care they have experienced by engaging stakeholders from disadvantaged communities would make the quality of care assessment more applicable to the expectations of users than that of health providers. By reviewing the medical records of respondents, the risk of recall bias is reduced, especially for measurement of the outcome variable.

## Abbreviations

ANC: Antenatal Care
CI: Confidence Interval
DoS: Department of Statistics
FANC: Focused Antenatal care
HEIS: Household’s Expenditure and Income Survey
JD: Jordanian Dinar
LMICs: Low and Middle Income Countries
MCH: Maternal and Child Health
MoPIC: Ministry of Planning and International Cooperation
OR: Odds Ratio
UNDP: United Nations Development Program
UNICEF: United Nations Children’s Fund
WHO: World Health Organization

## References

[1] Asamoah BO, Agardh A (2017). Inequality trends in maternal health services for young Ghanaian women with childbirth history between 2003 and 2014. BMJ Open.

[2] Aminur R, Monjura KN, Tahmina B, Sayem A, Nurul A, Iqbal A (2017). Trends, determinants and inequities of 4+ ANC utilisation in Bangladesh. J Health Popul Nutr.

[3] Bitew T, Hanlon C, Kebede E, Medhin G, Fekadu A (2016). Antenatal depressive symptoms and maternal health care utilisation: a population-based study of pregnant women in Ethiopia. BMC Pregnancy and Childbirth..

[4] Liu X, Behrman JR, Stein AD, Adair LS, Bhargava SK, Borja JB (2017). Prenatal care and child growth and schooling in four low- and medium-income countries. PLoS One.

[5] Kifle D, Azale T, Gelaw YA, Melsew YA (2017). Maternal health care service seeking behaviors and associated factors among women in rural Haramaya District, eastern Ethiopia: a triangulated community-based cross-sectional study. Reprod Health.

[6] United Nations Development Program (2011). A social determinants approach to maternal health:discussion paper.

[7] Lincetto O, Mothebesoane-Anoh, Gomez P, Munjanja S. Chapter 2: Antenatal care. In: Lawn J, Kerber K, editors. Opportunities for Africa’s newborns: practical data, policy and programmatic support for newborn care in Africa. Geneva: World Health Organization. 2006, 51–62. http://www.who.int/pmnch/media/publications/aonsectionIII_2.pdf. Accessed 22 June 2017.

[8] World Health Organization. WHO recommendations on antenatal care for a positive pregnancy experience. 2016.http://apps.who.int/iris/bitstream/10665/250796/1/9789241549912-eng.pdf. Accessed 20 February 2017. 28079998

[9] Pell C, Meñaca A, Were F, Afrah NA, Chatio S (2013). Factors affecting antenatal care attendance: results from qualitative studies in Ghana, Kenya and Malawi. PLoS One.

[10] Dowswell T, Carroli G, Duley L, Gates S, Gülmezoglu AM, Khan-Neelofur D, Piaggio G (2015). Alternative versus standard packages of antenatal care for low-risk pregnancy. Cochrane Database Syst Rev.

[11] Miltenburg AS, van der Eem L, Nyanza EC, van Pelt S, Ndaki P, Basinda N, et al. Antenatal care and opportunities for quality improvement of service provision in resource limited settings: a mixed methods study. PLoS One 2017; 12(12): e0188279. https://doi.org/10.1371/ journal.pone.0188279. 10.1371/journal.pone.0188279 PMC5728494 29236699

[12] Muhwava LS, Morojele N, London L (2016). Psychosocial factors associated with early initiation and frequency of antenatal care (ANC) visits in a rural and urban setting in South Africa: a cross-sectional survey. BMC Pregnancy and Childbirth.

[13] Low P, Paterson J, Wouldes T, Carter S, Williams M, Percival T (2005). Factors affecting antenatal care attendance by mothers of pacific infants living in New Zealand. N Z Med J.

[14] Abbas AA, Walker GJ (1986). Determinants of the utilization of maternal and child health services in Jordan. Int J Epidemiol.

[15] Obermeyer CM, Potter JE (1991). Maternal health care utilization in Jordan: a study of patterns and determinants. Stud Fam Plan.

[16] Shakhatreh FM, Abbas AA, Issa AA (1996). Determinants of infant mortality and the use of maternity services in Jordan. Dirasat medical and biological sciences.

[17] Alkhaldi SM (2016). Predictors of antenatal care utilization in Jordan: findings from a national survey. Jordanian medical. Journal.

[18] Department of Statistics [Jordan] and ICF International. Jordan population and family health survey 2012. 2013. https://dhsprogram.com/pubs/pdf/FR282/FR282.pdf. Accessed 15 July 2017.

[19] United Nations Development Program (2013). Jordan poverty reduction strategy: final report.

[20] United Nations Development Program, Department of Statistics, and Ministry of Planning and International Cooperation. Thinking differently about the poor “Findings from Poverty Pockets Survey in Jordan”. 2012. http://www.undp.org/content/dam/jordan/docs/Poverty/Jordan_Poverty%20Pocket%20Report.pdf. Accessed 16 July 2017.

[21] United Nations Children's Fund (2016). Analyzing equity in health utilization and expenditure in Jordan with focus on maternal and child health services.

[22] The Higher Health Council. The national strategy for health sector in Jordan 2015–2019. 2016.http://www.hhc.gov.jo/uploadedimages/The%20National%20Strategy%20for%20Health%20Sector%20in%20Jordan%202015-2019.pdf. Accessed 19 July 2017.

[23] Byrd TL, Law JG (2009). Cross-border utilization of health care services by United States residents living near the Mexican border. Rev Panam Salud Publica. Pan Am J Public Health.

[24] World Health Organization. Antenatal care in developing countries: promises, achievements and missed opportunities. 2003. http://apps.who.int/iris/bitstream/handle/10665/42784/9241590947.pdf?sequence=1. Accessed 19 July 2017.

[25] Andersen RM (1995). Revisiting the behavioral model and access to medical care: does it matter?. J Health Soc Behav.

[26] Babitsch B, Gohl D, Re-revisiting Andersen v LT. S behavioral model of health services use: a systematic review of studies from 1998–2011. GMS. Psychosoc Med. 2012; 10.3205/psm000089, URN: urn:nbn:de:0183-psm0000891. 10.3205/psm000089 PMC3488807 23133505

[27] World Health Organization. Standards for Improving Quality of Maternal and Newborn Care in Health Facilities. 2016. http://www.who.int/maternal_child_adolescent/documents/improving-maternal-newborn-care-quality/en/. Accessed 20 February 2018.

[28] De Masi S, Bucagu M, Tunçalp Ö, Pablo Peña-Rosas J, Lawrie T, Oladapo OT (2017). Integrated person-centered health care for all women during pregnancy: implementing World Health Organization recommendations on antenatal care for a positive pregnancy experience. Global Health Sci Pract.

[29] Bruce J (1990). Fundamental elements of the quality of care: a simple framework. Stud Fam Plan.

[30] Davidson PL, Andersen RM, Wyn R, Brown ERA (2004). Framework for evaluating safety-net and other community-level factors on access for low-income populations. Inquiry.

[31] Moore N, Blouin B, Razuri H, Casapia M, Gyorkos TW (2017). Determinants of first trimester attendance at antenatal care clinics in the Amazon region of Peru: a case-control study. PLoS One.

[32] Birmeta K, Dibaba Y, Woldeyohannes D (2013). Determinants of maternal health care utilization in Holeta town, Central Ethiopia. BMC Health Serv Res.

[33] Peer N, Morojele N, London L (2013). Factors associated with contraceptive use in a rural area in western Cape Province. S Afr Med J.

[34] Simkhada B, Teijlingen ER, Porter M, Simkhada P. Factors affecting the utilization of antenatal care in developing countries: systematic review of the literature. J Adv Nurs 2008; 61(3):244–260. doi: 10.1111/j.1365-2648.2007.04532.x PMID: 18197860. 10.1111/j.1365-2648.2007.04532.x 18197860

[35] Al-Qutob R, Mawajdeh S, Bin Raad F (1996). The assessment of reproductive health services: a conceptual framework for prenatal care. Health Care Women Int.

[36] Association of Reproductive Health Professionals (2008). Breaking the contraceptive barrier: techniques for effective contraceptive consultations.

[37] Asfawosen A, Mussie A, Huruy A, Wondeweson T (2014). Factors associated with maternal health care services in Enderta District, Tigray, northern Ethiopia: a cross sectional study. Am J Nurs Sc.

[38] Erlindawati CJ, Isaranurug S (2008). Factors related to the utilization of antenatal care services among pregnant women at health centers in Aceh Besar district, Nanggroe Aceh Darussalam province, Indonesia. J public health Dev.

[39] Nwaeze IL, Enabor OO, Oluwasola TA, Aimakhu CO (2013). Perception and satisfaction with quality of antenatal care services among pregnant women at the university college hospital, Ibadan. Nigeria Ann Ibd Pg Med.

[40] Ejigu T, Woldie M, Kifle Y (2013). Quality of antenatal care services at public health facilities of Bahir-Dar special zone, Northwest Ethiopia. BMC Health Serv Res.

[41] Nikie’ma B, Beninguisse G, Haggerty JL (2009). Providing information on pregnancy complications during antenatal visits: unmet educational needs in sub-Saharan Africa. Health Policy Plan.

[42] Anya S, Hydara A, Jaiteh L (2008). Antenatal care in the Gambia: missed opportunity for information, education and communication. BMC Pregnancy and Childbirth..

[43] Magoma M, Requejo J, Merialdi M, Campbell O, Cousens S (2011). How much time is available for antenatal care consultations? Assessment of the quality of care in rural Tanzania. BMC Pregnancy and Childbirth..

[44] Ndwiga C, Warren CE, Ritter J, Sripad P, Abuya T (2017). Exploring provider perspectives on respectful maternity care in Kenya: “work with what you have”. Reprod Health.

[45] Sharan M, Valente TW (2002). Spousal communication and family planning adoption: effects of a radio drama serial in Nepal. Int Fam Plan Perspect.

